# An Attentional Effect of Musical Metrical Structure

**DOI:** 10.1371/journal.pone.0140895

**Published:** 2015-11-23

**Authors:** Jonah Katz, Emmanuel Chemla, Christophe Pallier

**Affiliations:** 1 West Virginia University, Morgantown, WV, USA; Institut Jean-Nicod, CNRS, EHESS, IEC, Paris, France; 2 Laboratoire de Sciences Cognitives et Psycholinguistique, CNRS, EHESS, IEC, Paris, France; 3 INSERM-CEA Cognitive Neuroimaging Unit, U992, Neurospin Center, Gif-sur-Yvette, France; UNLV, UNITED STATES

## Abstract

Theories of metrical structure postulate the existence of several degrees of beat strength. While previous work has clearly established that humans are sensitive to the distinction between strong beats and weak ones, there is little evidence for a more fine grained distinction between intermediate levels. Here, we present experimental data showing that attention can be allocated to an intermediate level of beat strength. Comparing the effects of short exposures to 6/8 and 3/4 metrical structures on a tone detection task, we observe that subjects respond differently to beats of intermediate strength than to weak beats.

## Metrical structure and attention

**Fig 1:**
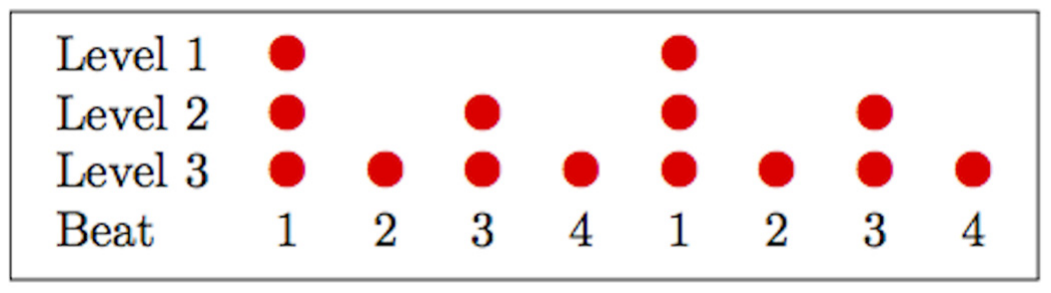
*Common time* in grid notation (showing two cycles).

According to music theory, isochronous (or near-isochronous) musical pieces possess a metrical structure which can be described by a grid indicating the relative prominence associated with various equally-spaced points in time, or *beats* [1]. Each beat on the horizontal axis is associated with some level of abstract prominence, indicated by the number of dots on the vertical axis. For example, Fig 1 shows the *grid* notation of a very frequent metrical pattern, known as *common time* or $\frac{4}{4}$. In this metrical pattern, every second beat is stronger than the ones preceding and following it, and every second one of those strong beats is stronger than the strong beats that precede and follow it. Common time is thus based on a pattern of four beats, in which the first one is the strongest beat, the second and fourth are the weakest, and the third is intermediate. More generally, a beat is stronger than any other beat associated with positions at fewer levels of the grid.

The strong beats correspond to prominent temporal positions which are often associated with musical events having greater duration, intensity or harmonic information (which we will refer to collectively as *accent*) [1]. Accordingly, listeners find it easier to memorize and replicate sequences of sounds where accents correspond to strong metrical positions [2]. A listener’s facility at inferring specific metrical structures from specific accent patterns is influenced by their culture-dependent history of exposure to those patterns [3]. Non-auditory properties such as synchronized movement can also contribute to the perception of accent [4].

A number of empirical studies show that listeners distinguish at least two levels of metrical prominence [5–8]. None of these studies, however, conclusively show that listeners infer hierarchical metrical structures of the type shown in Fig 1.

In particular, no study has demonstrated that metrical strength relations exist between intermediate levels in the grid. Most previous studies on the topic have either investigated: (1) the difference between beats at the *highest level in the grid and all other levels*, or (2) the difference between *beats and non-beats*. The sole previous study of which we are aware that had the potential to examine differences between beats of intermediate strength [5] does not report the relevant analyses. Below we offer a brief review of some relevant studies and ways to explain their results without assuming multiple levels of hierarchical prominence in the metrical grid.

### Strongest beats vs. other beats

We review in this section several studies showing that subjects attend or react differently to the strongest beat than they do to all other beats. Generally, the ‘other’ beats at sub-levels of metrical strengths are not further distinguished in these studies.

Repp [9], using an oddball detection task, shows that subjects are most sensitive to acoustic differences that occur on the first (strongest) beat of a repeating triple metrical pattern. The interpretation is that subjects allocate more auditory attention to these beats. Using a tapping task, Patel et al. [8] show that subjects deviate from pure isochrony in a qualitatively different way on the strongest level of beat from the way they perform on all other levels: in particular, it appears that subjects anticipate (i.e., tap ‘too early’) less on the strongest beat of an implied quadruple meter than they do on other beats. Dawe et al. [10] show that subjects produce coherent judgments about which event in an isochronous series of events represents the strongest level of beat, and that these decisions are affected by harmonic properties of the stimulus.

The most relevant study for our purposes is the one described by Palmer et al. [5]. They show that in a sequence of repeating, isochronous ‘reference’ events, a ‘probe’ event is judged as more well-formed when it occupies a temporal position that would be relatively strong in a meter defined by the reference events. The authors manipulated the induced metrical structure by manipulating the distance of the reference events, as they show with a corpus study that this correlates with metrical structure (roughly: slower rhythms correspond to more sub-divided metrical structures).

This design has, in principle, the potential to uncover multiple levels of hierarchical metrical prominence; the relevant comparisons, however, are not implemented. The authors show that there is a main effect of probe position, but do not carry out pairwise comparisons between levels of hypothesized metrical strength. The visual presentation of their data suggests that some subjects in some conditions display a trend towards distinguishing between beats of intermediate strength, in particular the well-formedness judgments from trained musicians in $\frac{3}{4}$ and $\frac{6}{8}$ time, but this pattern is far from clear.

Povel and Essens [2] find that subjects are able to memorize and reproduce rhythmic sequences with fewer repeated listenings when those sequences reinforce strong positions in the metrical structure through the presence of auditory events, especially psychoacoustically salient (accented) events, as opposed to sequences where fewer auditory events corresponded to strong metrical positions. The (rather successful) algorithm used to predict the amount of reinforcement for a particular metrical pattern is based on whether or not positions corresponding to the strongest beat in that pattern (the periodicity of the temporal clock, in their terms) are filled by auditory events. But the algorithm does not distinguish between sub-levels of metrical strength.

These studies provide abundant evidence for a privileged status of strong metrical positions. This is a necessary but not sufficient condition for establishing that subjects infer a metrical structure with more than 2 levels of prominence.

### On beat vs. off beat distinction

Several studies associated with the Dynamic Attending framework [11], which attempts to model the fine structure of musical meter and pulse as modulation of attention, show that subjects distinguish between temporal positions strictly below the level of the strongest beats. However, they do not show a distinction between sub-levels of metrical structure of different relative strengths, rather they show a distinction between on-beat and off-beat events (or alternatively, isochronous and non-isochronous sequences of events). Such a distinction is expected even in the absence of a hierarchical organization of the on-beat events.

Jones [6], for instance, find that subjects are more accurate on a relative-pitch judgment task for target events that fall on a (relatively high-level) metrical beat than for events that do not fall on a metrical beat (or, equivalently, fall on a beat associated with such a small subdivision of the grid that listeners are incapable of constructing such a representation). Penel and Jones [12] report similar results for detecting an oddball pitch, with more false alarms for events that do not fall on a beat, as well as faster response times in the absence of perturbation of isochrony, i.e. on beats.

Jongsma et al. [7], like Palmer et al. [5], use well-formedness judgments as a proxy for metrical strength given particular preceding contexts. They show that subjects judge target sounds in temporal positions that were occupied by sounds in a preceding context as more well-formed than target sounds in temporal positions that were not occupied by sounds in the preceding context. This effect is more pronounced for trained musicians. There is also limited evidence that trained musicians prefer target sounds on temporal positions that subdivide beats in the preceding context by two over smaller subdivisions.

These studies demonstrate that subjects allocate more attention to events that fall on a beat in the metrical grid than events that don’t. Again, this is a necessary but not sufficient condition for concluding that listeners infer hierarchical metrical grids. While the results discussed in the previous section did not offer evidence in favor of a hierarchical organization with more than 2 levels, these results do not provide evidence in favor of the existence of intermediate levels of strength *within* the grid.

One may argue that the difference between the strong-beat vs. weak-beat distinction discussed above and the on-beat vs. off-beat distinction is only one of temporal scale. For instance, if stimuli reinforce a periodicity of 800 ms, and subjects perform badly when a target occurs 50 ms away from the established pulse, we could either say that target falls on a non-beat or a weak beat at the 16th-note level with regard to the established pulse. However it seems *a priori* unlikely that subjects are constructing a grid of infinite depth or with beats 50 ms apart [13].

### Subdivision by small integers

All of the results discussed here are consistent with a binary strong/weak beat distinction coupled with (or equivalent to) a binary on-beat/off-beat distinction. Because several influential theories of meter share the prediction that metrical strength should be cumulative and hierarchical [1, 6, 11, 14], it seems to us important to try to test this prediction in as rigorous a manner as possible. One purpose of the current experiment, then, is to examine whether subjects consistently differ in their tendency to attend to events occurring on beats of intermediate strength in a metrical grid. This requires two positions that are both beats, neither of which is the strongest beat in a given pattern. The current experiment also attempts to avoid a confounding factor that could give the appearance of hierarchical meter even if subjects were not inferring such structures: the problem of small-integer subdivision.

Even if we could show that, in the context of a quadruple meter like that in Fig 1 above, subjects attend more readily to events on the third beat than they do to events on the fourth and second, it would not necessarily be evidence for a hierarchy of beat strength. An alternative, and perhaps simpler, hypothesis is that subjects infer one periodicity, and have a limited capacity to subdivide that period into equal parts. Subdividing into a small number of parts (e.g. 2) should incur less cognitive ‘cost’ than subdividing into a large number of parts (e.g. 3). Any task that involves attending, reacting, or remembering an event on beat 3 requires only a subdivision into 2 equal parts; beats two and four would both require subdivision into 4 parts. Lower levels of beat strength and off-beat events would require subdivision into an ever-greater number of equal parts, which would eventually become practically impossible. There is already limited evidence that an advantage for even subdivisions cannot be the whole story about meter: Hannon & Trehub [3] fail to find any processing or memory advantages for metrical structures with even subdivisions over those with uneven subdivisions for subjects enculturated with Balkan odd-meter music or those who have not been enculturated yet, i.e., infants. Of course, it is hard to draw firm conclusions from a negative result, so we nonetheless use the small-integer approach as a kind of null hypothesis here.

In the current experiment, we show that reversing the relative metrical strength of two positions X and Y reverses the direction of reaction-time differences at these positions X and Y. Crucially, the positions of X and Y with regard to the strongest beats remain constant; thus the small-integer explanation and other ‘absolute position’ explanations are ruled out.

### The current study

**Fig 2:**
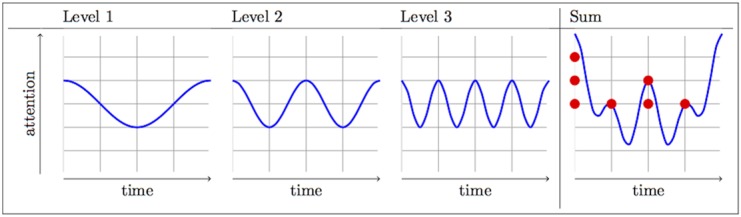
Toy model of attention for *common time* using sine oscillators.

Our method of investigating listeners’ mental representations of meter is suggested by [14]. Working in the framework of Dynamic Attending Theory [11], they propose an interpretation of metrical prominence in terms of attention. Different levels of metrical structure correspond to hierarchically coupled attentional oscillators. This model and Large and Palmer’s [15] extension of it are actually far more complex than what is necessary for the current study; this is because the original models are meant to deal with a listener’s response to perturbations in isochrony, which are not investigated here. Instead, we describe a greatly simplified version of those previous models without any corrections for phase irregularity. This is quite similar to the simpler model presented by Jongsma et al. [7]. For instance, if we use sine oscillators for simplicity, the three-level common-time pattern in Fig 1 would be interpreted as three oscillators, one at each level, with attention at any point in the auditory stream corresponding to the addition of the three waves. This is shown in Fig 2

Put simply, increased metrical prominence entails increased attention on the part of the listener. If this interpretation of metrical prominence is correct, it will be possible to observe the effect of hierarchical structure on tasks that require attention, such as detecting a sound in a given metrical background. It is still an open question whether metrical attention should aid in these tasks (by focusing attention in the ‘right’ place) or hinder them (by using attentional resources that are consequently rendered unavailable for other tasks). See the paragraph on predictions in the methods section for details. The use of reaction time as a proxy for attention, or for specific types of expectancy that resemble the Dynamic Attending conception of attention, is fairly common in the music-perception literature [12, 16–18].

**Fig 3:**
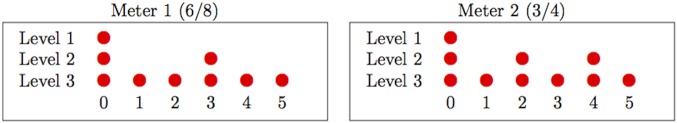
Main meters from the experiment.

To provide convincing evidence that subjects’ attention is modulated by metrical structure, regardless of preferences for subdivision by small integers, one must dissociate metrical strength and subdivision by small integer. We propose to do so by studying and comparing duple and triple meters of the kinds presented in Fig 3.

In both meters, beats 2 and 4 are located at one and two *thirds* of the sequence (subdivision by 3), while beat 3 is in the *middle* of the sequence (subdivision by 2). In meter 1, beat 3 is accordingly stronger than beats 2 and 4. But crucially, in meter 2, metrical structure and subdivision by small integers depart: beat 3 is weaker than beats 2 and 4. The results from the following experiment will show that attention tracks metrical structure rather than with subdivision by small integers, by revealing an attentional effect that depends upon the weak/strong distinction rather than the division by 2/3 distinction (or any other property that would align with linear position in a string of sounds). Because they involve differentiating various beats of intermediate strength, these results also provide evidence for the hierarchical nature of metrical structure.

## Experiment

**Fig 4:**
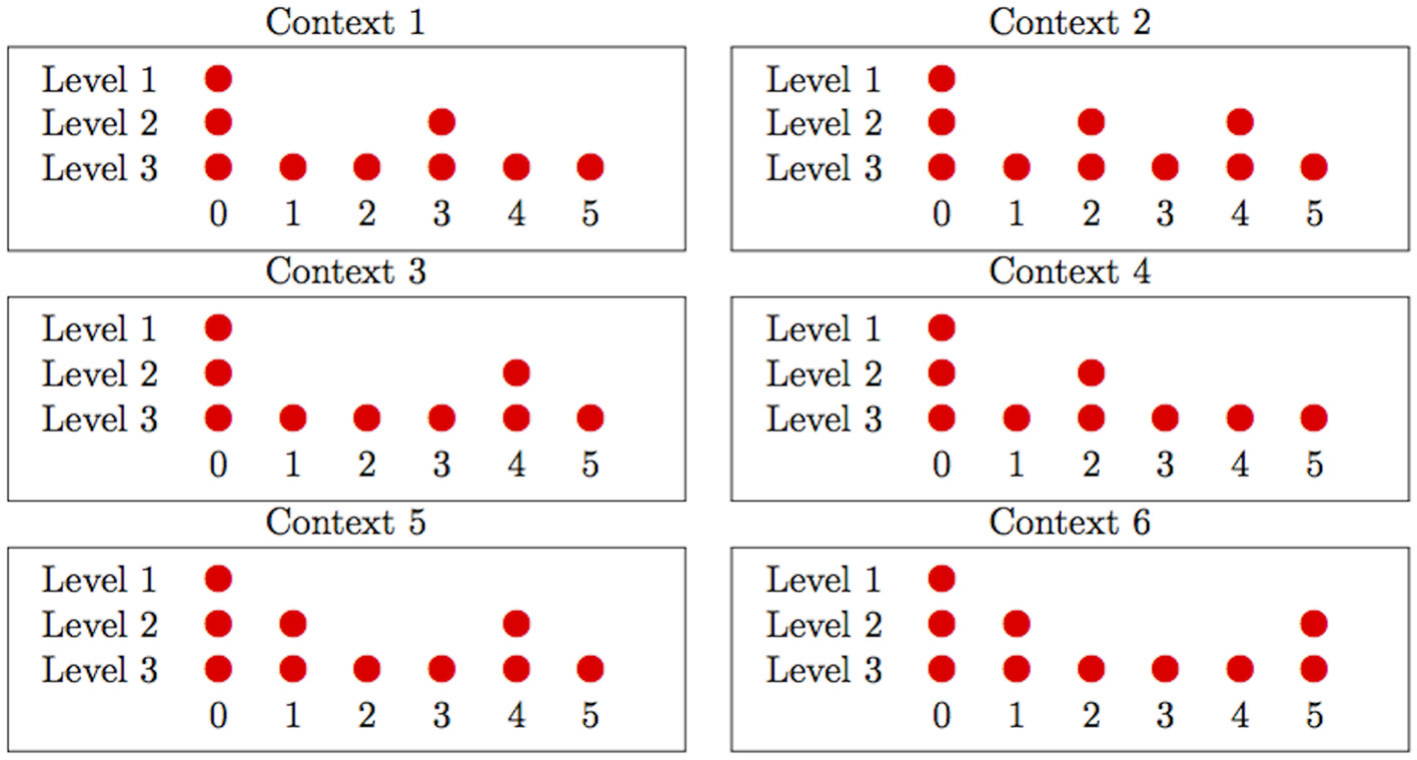
The six *Contexts* used in the experiment. Note that Contexts 1 and 2 correspond to the metrical patterns 1 and 2 displayed on Fig 3.

Subjects listened to sequences of cymbal sounds modulated in amplitude to match one of the patterns described in Fig 4. Henceforth, we refer to these patterns as *Contexts*.

Each stimulus corresponded to a repetition of 6 cycles of such patterns. In one of the last two cycles, one of the cymbal sounds was replaced by a high pitch tone. The task of the subject was to respond to this tone, pressing a response button as fast as possible. The test tone could occur with equal probability in any of the beat positions 1–5.

Our hypotheses were the following: (i) Contexts 1 and 2 robustly reinforce particular metrical structures, while Contexts 3 to 6 do not, or do so less robustly, and (ii) metrical structures induce an attentional effect, while subdivision by small integers does not (or does so to a smaller extent). Focusing on beats 2, 3 and 4, we thus predict that Contexts 1 and 2 will show *opposite* patterns of reaction-times on these beats. The less metrical Contexts 3 to 6 are included as controls; we predict that they will not show the same metrical effects as contexts 1 and 2, and we also use these Contexts to test the idea that subjects are simply reacting to levels of expected loudness.

The reason why contexts 3-6 should not induce metrical structure as robustly as 1 and 2 is because each of these accentual patterns is ambiguous with regard to what type of metrical structure it is most consistent with. In particular, Contexts 3 and 4 are broadly similar to 2, and might be expected to reinforce the same 3/4 metrical structure, but each Context is missing an accent that would be expected on a strong beat if 3/4 were being reinforced. Either one of these Contexts could alternately be interpreted as reinforcing a 6/8 meter like pattern 1, but with an accent occurring one beat earlier or later than the stronger beat in position 3, where it would be expected. Contexts 5 and 6 are also broadly consistent with either 3/4 or 6/8 meter, but no matter what metrical pattern is assigned to these patterns, they necessarily show a reversal of normal relationships between accents and metrical strength: so, for instance, if a listener decides that Context 6 is unfolding in 3/4 meter, the listener will find that position 1 is associated with a stronger accent than position 2, even though position 2 is associated with a stronger beat. This discussion assumes that subjects will infer a typical metrical structure in these cases, where beats at a given level are evenly-spaced, and then find that the accentual pattern doesn’t perfectly match the structure they’ve inferred. There are, of course, other possibilities: listeners may infer a ‘defective’ metrical structure, i.e., one where beats at a given level are not evenly spaced in time but instead correspond to the location of accents; or listeners may simply fail to assign any metrical structure to these contexts (or fail to infer higher levels of structure). Either way, the prediction is that behavioral reflexes of metrical structure induction will be weaker, more variable, or less likely to be present in Contexts 3-6 than in Contexts 1-2, which unambiguously reinforce particular metrical structures.

### Method

#### Participants

Twenty subjects participated in the experiment, ranging in age from 23 to 39 year old, with 9 males and 11 females. They reported having no musical training (beyond casual listening to music). They were paid 5 euros for their participation. This research, and the consent forms subjects signed, were approved by the Conseil dâ??Ã©valuation ethique pour les recherches en sante (CERES).

#### Stimuli

Two sounds were used to generate the stimuli: a natural recording of a cymbal sound, edited to fit within 80 ms, and a pure tone of 880Hz lasting also 80 msec. The inter-onset-interval in all stimuli was 200 ms.

The accentuation patterns described in Fig 4 were created by using copies of the cymbal sound modulated in amplitude by factors of 1, 0.75 and 0.5 (corresponding to strongest, intermediate and weakest beats respectively).

In each trial, 6 cycles of one accentuation pattern, or Context, were played. In the 5th or 6th cycle, one of the cymbal sounds in position 1-5 was replaced by the pure tone.

#### Procedure

Participants were given the following instructions (in French): “You are going to hear some rhythms. Your task is to listen to these rhythms and push the space bar as fast as possible when you hear the beep.”

The experiment was controlled by a program written in Python and using the library pygame (www.pygame.org). The stimuli were generated on-line, played to the participant and the response time, measured from the onset of the target tone, was recorded at each trial. The computer waited for the completion of the stimuli and for the subject’s response before proceeding to the next trial after a 1 second delay.

Each participant received a total of 180 trials: 3 repeated measures for a given Context (1-6), a given beat position (1-5) and a cycle position (beat played either during the 5th or during the 6th repetition of the pattern). The trials were administered in random order. The whole experiment lasted about 30 minutes.

#### Predictions

Increased metrical prominence should entail increased attention on the part of the listener. If our stimuli induce a metrical structure, performance at the detection task should be modulated by attention, and consequently by the metrical prominence at the position where the probe appears. More precisely, one may expect that performance at the detection task will be increased at strong metrical positions. This prediction is in line with effects reported by Jones [6] and Repp [9] for judgments of relative pitch and intensity, respectively. However, a second type of effect is observed for detection tasks, referred to as ‘capture effects’ by Penel and Jones [12]. These authors found that in a tone detection task, some subjects in some conditions were *slower* on beats than non-beats. This appears to hold true for a subset of subjects even when the metrical position of a target tone is not task-relevant. The authors suggest that this is a kind of *reactive* attending, where the occurrence of an event in an unexpected temporal position renders it more salient. Hence, one may expect that performance on a detection task may *decrease* with structural prominence of the position, as Penel and Jones [12] found in several detection studies. Alternatively, our task may not induce metrical structures at all, or we may find an effect of absolute temporal (rather than structural) position on attention.

### Results

**Fig 5:**
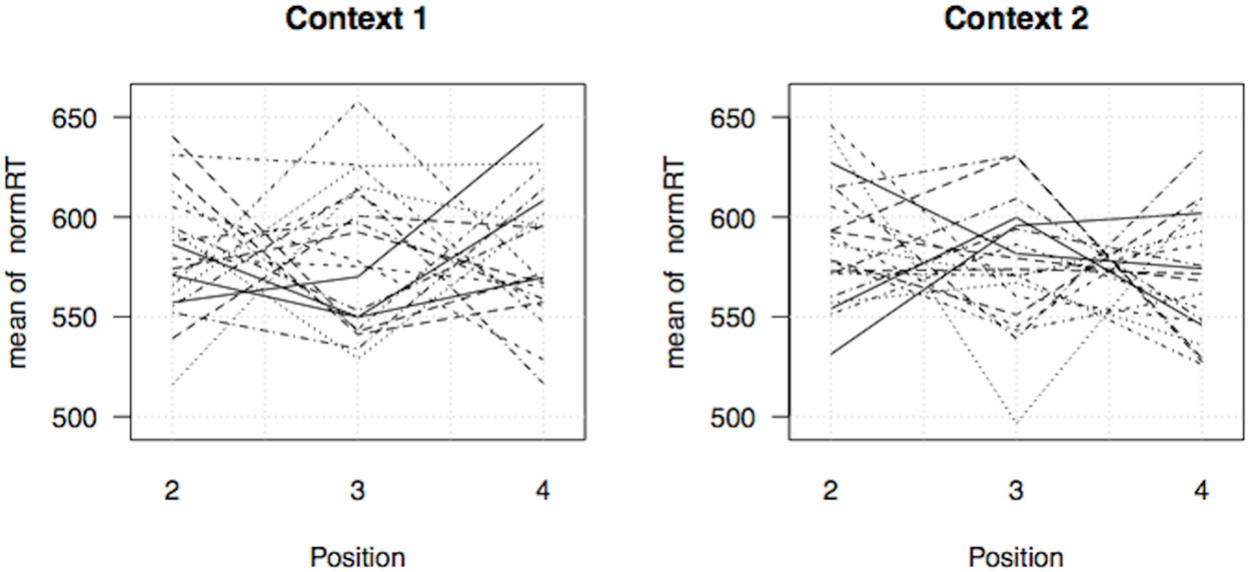
Reaction times for central beats (Positions 2, 3 and 4) for the first two Contexts (each line corresponds to one participant; RTs were normalized by removing the main effects of participants).

After removing responses below 200 ms or above 1500 ms, we computed each participant’s average reaction times (RT) as a function of Position (1 to 5) and Contexts (1 to 6). Fig 5 displays the most critical subset when the target tone occurred in Positions 2, 3 or 4 following the inducing Contexts 1 or 2. On this graphics, no obvious general pattern distinguishes Positions within each Context, and indeed a two-way anova (analysis of variance) on the Log transformed RT, with factor Context (1 vs. 2) and Position (2, 3 or 4) reveals that the interaction between the two factors is not statistically significant *F*(4, 76) = .58. Moreover, within each Context, the main effect of position is also non significant (*F*(2, 38)<1.3).

Closer inspection of Fig 5, however, suggests that individual patterns are not flat, but seem to have ‘V’ or inverted ‘V’ shapes. We therefore conducted post-hoc tests of the hypothesis that different participants may show attentional effects in different directions, some subjects being faster on strong beats and others on weak beats. Recall that it is not clear in which *direction* one would expect such an attentional effect to go: we could either see facilitation (acceleration) or inhibition (slow down) on strong beats (see the ‘Predictions’ section).

We used the difference between response times on strong beats and response times on weak beats as an index of the attentional effect. For each subject, we computed the difference between the middle, strong beat and the surrounding, weak beats for Context 1: $V_{1}={\text{RT}}_{3}-\frac{1}{2}\left({\text{RT}}_{2}+{\text{RT}}_{4}\right)$, in which *RT*_*n*_ represents the mean response time on beat *n*. A positive *V*_1_ indicates that the subject is slower to detect a target occurring on beat 3 than on beats 2 and 4.

**Fig 6:**
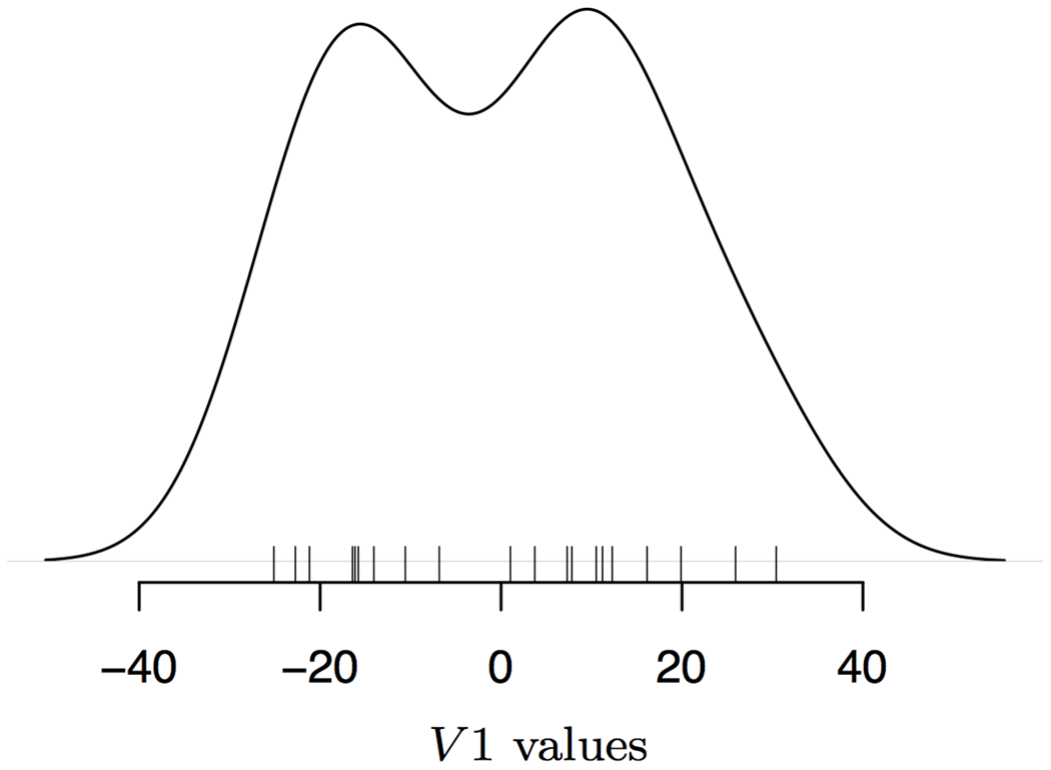
Estimated Probability Density of *V*1, the RT difference between Strong and Weak beats, in Context 1. NB: bandwidth set by default to 8 for the representation.

**Fig 7:**
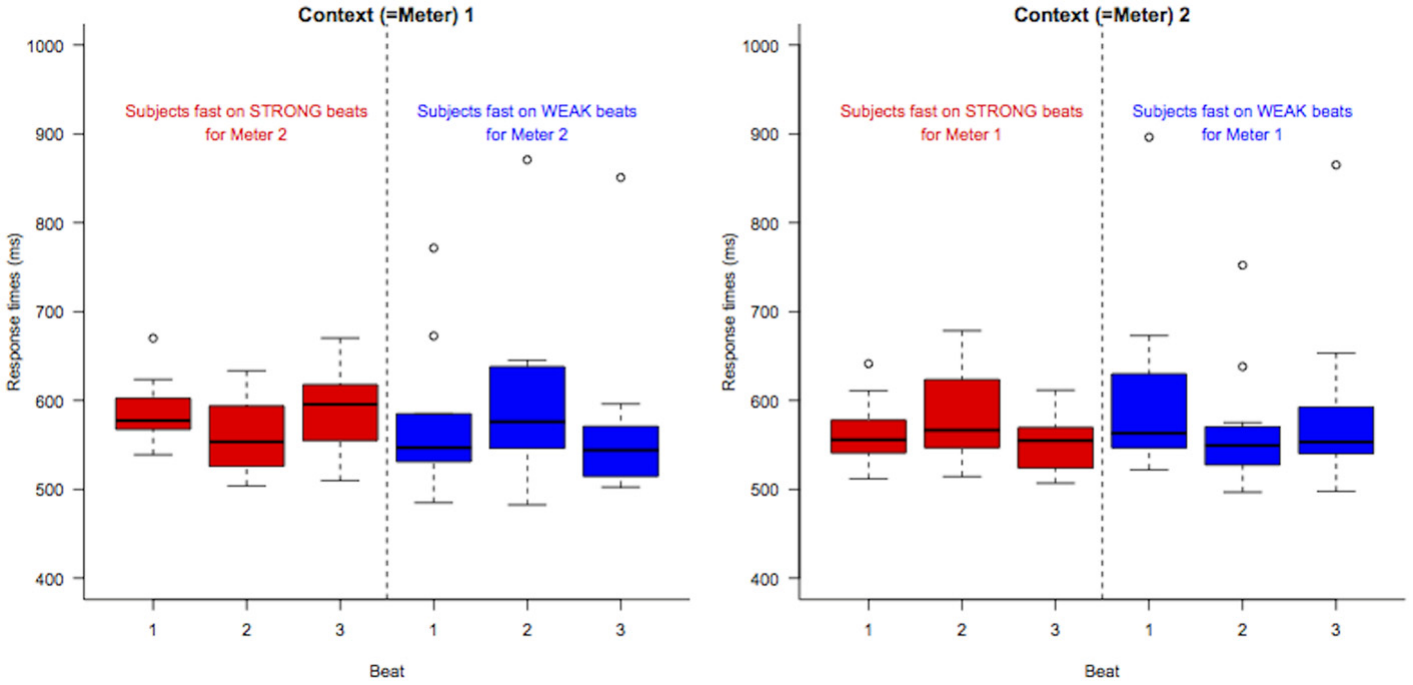
Boxplots of (untransformed) response times for central beats in Contexts 1 and 2, when participants are split according to their behavior for the other Context.

**Fig 8:**
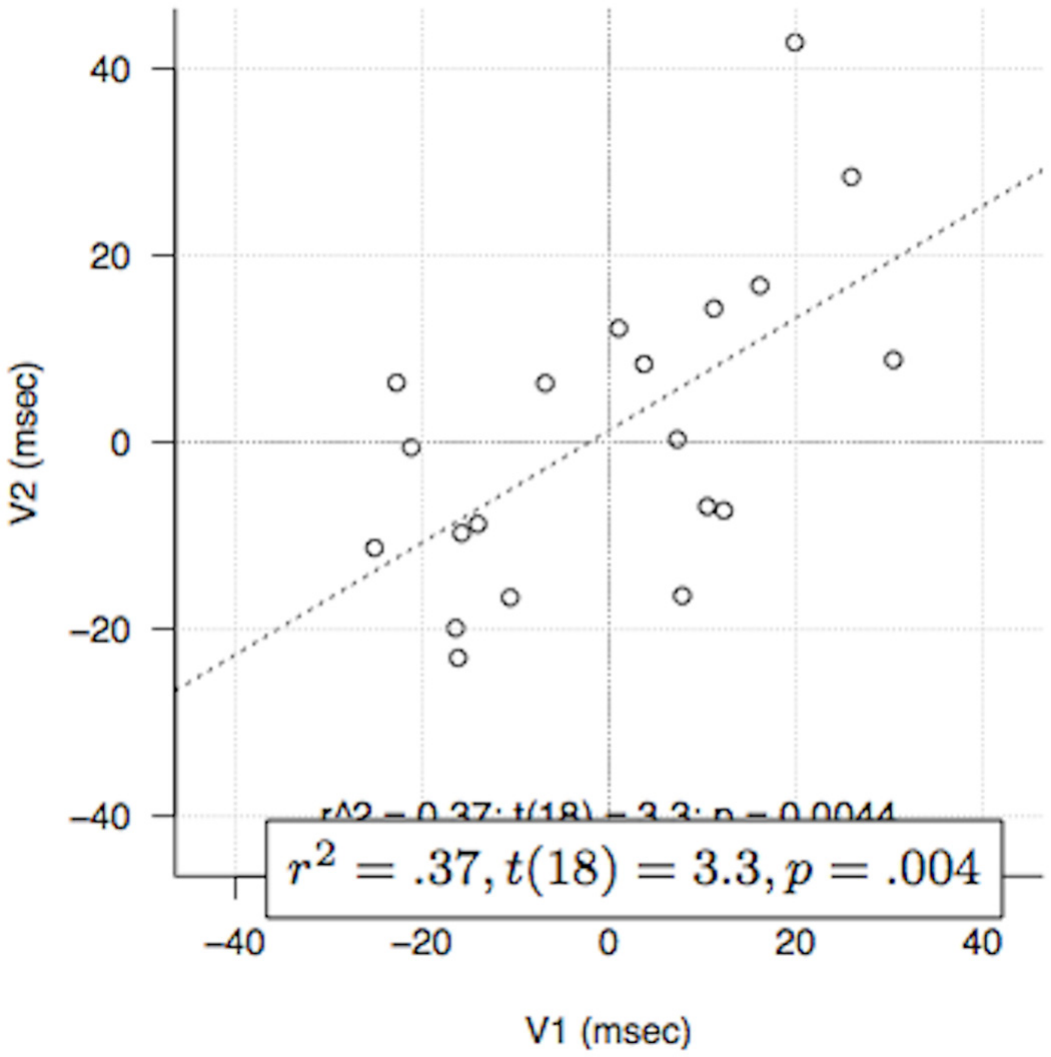
Correlation of the Strong *minus* Weak measure for well-formed Context 1 (*V*1) and Context 2 (*V*2).

Fig 6 shows the distribution of *V*_1_ in Context 1. We simply note that the overall distribution of this measure looks bimodal, as if some participants were faster on strong beats and some on weak beats. We will not assess this mere bimodality with statistical scrutiny. Crucially, however, we note that the attentional effect should be consistent within subject and *across* meters. A relevant test compares behavior on strong and weak beats for Context 1 to a corresponding measure of the difference between strong and weak beats for Context 2 ($V_{2}=\frac{1}{2}\left({\text{RT}}_{2}+{\text{RT}}_{4}\right)-{\text{RT}}_{3}$). We found that, indeed, participants with positive *V*_1_ have higher *V*_2_ than participants with negative *V*_1_ (*t*(17) = 2.9, *p* <.02). Similarly, participants with a positive *V*_2_ have higher *V*_1_ than participants with negative *V*_2_ (*t*(17) = 2.6, *p* <.02). Concretely, Fig 7 shows the response times for each of the central beat for Contexts 1 and 2, with participants split based on their behavior on the other context. This figure shows that participants are either fast or slow on strong beats, but behave (almost) consistently across Contexts 1 and 2. Moreover, as shown in Fig 8, there is a robust correlation (*r* ^2^ = .37, *t*(18) = 3.3, *p* <.01) between the effects found on Context 1 (*V*_1_) and Context 2 (*V*_2_). This figure shows that the attentional effect takes different forms for different participants (most of the participants fall either in the upper right quadrant or in the bottom-left quadrant), but is consistent across Contexts. Subdivision by small integers, on the other hand, would have led us to expect no such consistency: if the relevant factor were only subdivision, subjects would behave identically on beats in a given position across Contexts. The measure we have used here, which subtracts beat 3 from beats 2 and 4 in Context 1, but *vice versa* in Context 2, would then show a *negative* correlation.

#### Could it be a mere acoustic effect?

The hierarchical strength of a beat in our experiment is confounded with the acoustic strength of its realization as a cymbal sound. One objection, then, is that the results above may be purely acoustic, rather than reflecting induced metrical structure. This explanation is unlikely for three reasons.

1. On the beat on which the target high-pitched tone was played, there was no cymbal sound. Hence, there was no immediate difference in the stimulus that could drive an acoustic effect. One may wonder instead whether the *expectation* of hearing a relatively loud or soft cymbal sound in the target position could drive the effect; Jongsma et al. [7], for instance, entertain such a ‘sequential’ model before concluding that it cannot account for their data. For completely regular and evenly subdivided accent patterns such as our contexts 1 and 2, this sequential model would be virtually indistinguishable from what we refer to as metrical structure; for the less regular and even contexts 3–5, this ‘sequential’ theory appears to make the wrong predictions, as discussed below. A refined version of this objection is that the effect could be driven by the *preceding* beat. This would not, however, explain the correlation found for Contexts 1 and 2. If beats are categorized based on the relative loudness of the preceding beat, beats 2-4 would be weak-weak-strong for Context 1 and weak-strong-weak for Context 2, which would predict no correlation or a correlation in the opposite direction of what we found. Note as well that Contexts 2 and 4 would be fully identical in this respect (soft-loud-soft), but we found no reliable correlation of the kind presented in Fig 8 between these meters (*t*(18)<.17, ns).
2. Consider Contexts 3 to 6. They do not correspond to well-formed meters, and as such should not induce any particular metrical effect. This means that the differences in loudness in these patterns are *only* loudness differences, rather than hierarchical metrical differences. Hence, a positive Strong-Weak correlation (or Loud-Soft correlation) involving one of these patterns would indicate that the effect we found was not due to metrical structure, but to superficial acoustic properties.
  We investigated all possible correlations for beats 2 to 4, i.e. correlations between strong-weak differences in any pairs of Contexts 1 to 5. Context 6 was omitted because it shows no strength/loudness differences between these beats. We found only a significant *negative* correlation between Context 1 and Context 5: subjects who were faster on loud beats in Context 1 were *slower* on loud beats in Context 5, and *vice versa* (*t*(18) = −3.0, *p* <.01). This effect would not be predicted by any of the hypotheses considered here. It may be that subjects’ mentally ‘repair’ defective Context 5 as some well-formed variant, but none of the obvious candidates for repairing the Context into a well-formed meter would produce such an effect. We leave it to future research to see whether this effect is replicable and determine the explanation, but it crucially does not challenge the interpretation we propose for our main effect. More importantly, no other of these correlations came close to significance.
3. Finally, note that Contexts 3 and 4 are identical to Context 2, except that one of the beats of level 2 has been ‘demoted’ to level 3. Focussing on the pair of beats (beats 2 and 3 or beats 3 and 4) for which Context 2 is realized acoustically similarly to Contexts 3 and 4, respectively, we find no correlation between the effect in Context 2 and its relevant counterpart. Again, we propose that this is because even though their acoustic realization is comparable, these other Contexts do not induce a proper metrical structure and thus do not generate the metrical effect found for Context 1.

### Discussion

One aspect of the results that remains unexplained is *why* subjects differ as to the direction in which metrical strength affects response time. We first note that we are not aware in the relevant literature on attention of attempts to analyze such a difference in RT *patterns* between participants. This makes the interpretation of the current result more challenging but also interesting as the first of its kind.

One possibility is that some subjects devote more cognitive resources to using hierarchical metrical structure as a cue to where beeps might occur; this strategy, incidentally, would fail in the current task, where only the lowest-level of pulse is useful in this regard. Other subjects may devote more cognitive resources to using the global properties of stimuli as a cue; this would be fairly fruitful in the current task, because as a stimulus gets longer and longer with no beep yet detected, the probability of the beep occurring on the next beat grows higher and higher. One would predict that subjects who are (implicitly or explicitly) keeping closer track of the hierarchical aspect of metrical structure might show faster responses on stronger beats, if their attention is ‘coupled’ to metrical structure to a greater extent. Subjects tracking global properties of the stimuli, on the other hand, might find metrical structure to be a *distraction* from the task, thus slowing down with increased metrical strength.

Although this explanation sounds promising, we can find no evidence in our data that it is correct. In particular, a subject’s general effect for strength vs. weakness across Contexts 1 and 2 does not appear to correlate in any way with the amount that subject speeds up in the second half of the possible target beats. It is possible that this measure is somehow unable to detect the global vs. local difference discussed above, or the explanation for the differences in effect direction may lie elsewhere.

## Conclusion

This experiment showed a genuine effect of hierarchical metrical structure. We showed that subjects’ attention differs between beats of intermediate prominence in a hierarchical meter, not merely between the strongest beat and others or between beats and non-beats. The effect is also not reducible to facilitation for beats falling at subdivisions by smaller integers. Coupled with previous results regarding the contrasts just mentioned, the data suggest that metrical structure is indeed cumulative and hierarchical, as suggested by metrical theories.

Recall the non-metrical subdivision hypothesis: subjects are more skilled at subdividing intervals into a smaller number of equal parts. If we had found simply that subjects respond differently to the events occupying beat 3 in Context 1 than the events occupying beats 2 and 4, for instance, this would not be evidence that they inferred a metrical grid where beat 3 is stronger than beats 2 and 4. Because beat 3 is exactly half-way between the strongest level of beat in the stimuli, this result may just show that subjects attend more closely to subdivisions of the most salient periodicity in the stimulus into two parts than they do to subdivisions into three parts. Similar reasoning would suffice to explain virtually all of the previous results in the metrical attending and entrainment literature.

The fact that subjects changed their manner of responding depending on the preceding context, attending to triple subdivisions of a higher-level unit over double subdivisions when that choice was reinforced by the Context, offers clear evidence that metrical attending goes beyond simple preferences for halves over thirds or larger-integer subdivisions. Subjects adjust their attention to various periodicities in the acoustic signal on the basis of inferred metrical prominence, not solely on the basis of ease of subdivision.

Although this prediction of metrical theory seems basic, it has been neglected in previous research. This may be in part because the subdivision theory is quite similar in most of its predictions to richer theories of metrical structure. The hypothesis that listeners infer hierarchical grids where each level is constrained to be evenly spaced makes rather similar empirical predictions to the hypothesis that listeners preferentially split intervals into a small number of component parts. Indeed, the metrical theory is an addition to, not a replacement for, the subdivision theory: nothing about the even spacing property of metrical theory dictates that a higher level beat is more likely to occur every two lower-level beats rather than every 5 or every 7. A second reason is that the subdivision theory may seem too simple to plausibly handle the rhythmic diversity of actual music. Music theorists have never seriously entertained the idea that preferential subdivision into halves is a theory of metrical structure all by itself. It is the simplest explanations, however, that ought to be ruled out first, and no prior study has demonstrated that intermediate levels of metrical structure between highest-level beats and lowest-level (equivalent to non-) beats exist, nor that listeners can be induced to attend to larger-integer subdivisions when smaller-integer subdivisions are also present in the stimulus.

Several questions remain for future research.

- First and foremost, we found that subjects differ as to whether metrical strength facilitates or inhibits an attentional task. We entertained one explanation involving a difference in global vs. local attention, but found no support for the specific aspects of the hypothesis.
- Another possible direction for future research concerns the notion ‘well-formed meter’, which exists in Lerdahl’s theory [1] and, to some extent, in Povel and Essens’ theory [2]. Research on affective and sensorimotor responses to rhythm suggests that they do not straightforwardly track the strength of metrical representations, but instead result from the interplay of several factors including the violation of metrical expectations [19, 20]. The current results, in contrast, suggest a criterion for detecting and quantifying the well-formedness of a meter. We found that contexts which unambiguously imply well-formed meters through differences in accent induced an attentional effect tracking the hierarchical structure of the corresponding meter. But contexts that imply less well-formed meters (e.g., because some position failed to be regularly accented) failed to induce a meaningful attentional effect. Hence, the magnitude and reliability of attentional effects that a given context induces may constitute a useful measure of the well-formedness of various underlying metrical structures implied by that context.
